# Combined low-intensity pulsed ultrasound and extracorporeal shock wave therapy reduces pain and inflammation in knee osteoarthritis patients

**DOI:** 10.1038/s41598-025-30807-7

**Published:** 2025-11-29

**Authors:** Fater A. Khadour, Younes A. Khadour, Osama Ibrahim Khouly, Xiuli Dao

**Affiliations:** 1 Department of Rehabilitation, Faculty of Medicine, Al Baath University, Homs, Syria; 2 Department of Physical Therapy, Health Science Faculty, Al-Baath University, Homs, Syria; 3 Department of Rehabilitation, Tongji Hospital, Tongji Medical College, Huazhong University of Science and Technology, 1095#, Jie-Fang Avenue, Qiaokou District, Wuhan, 430030 Hubei China; 4 Department of Physical Therapy, Cairo University, Cairo, 11835 Egypt; 5 Department of Sport Education, Neijiang Normal University, Sichuan, 641004 China

**Keywords:** Extracorporeal shock wave therapy, Knee osteoarthritis, Synovial fluid, Low-intensity pulsed ultrasound, Osteoarthritis, Rheumatoid arthritis, Osteoarthritis, Risk factors, Quality of life, Outcomes research

## Abstract

Knee Osteoarthritis (KOA) is a degenerative joint condition that leads to pain and limited mobility. Non-invasive treatments like Low-Intensity Pulsed Ultrasound (LIPUS) and Extracorporeal Shockwave Therapy (ESWT) help manage symptoms and support recovery. While both methods are effective, no studies have directly compared ESWT alone to its combination with LIPUS (LESWT, low-intensity pulsed ultrasound + ESWT) for KOA. This study aims to assess their efficacy and provide evidence for treatment choices. The study included 110 patients with KOA who underwent LESWT, forming the LESWT group, and another 110 KOA patients who were treated with ESWT, constituting the ESWT group. Evaluations were conducted to compare clinical outcomes, levels of inflammatory markers in joint synovial fluid, and the occurrence of adverse events before and after the treatment. The LESWT group showed a higher clinical effective rate (87.3%) compared to the ESWT group (73.6%, *p* < 0.01), with greater improvements in LKSS, Lequesne index, VAS, WOMAC, and ROM scores (*p* < 0.05). Levels of inflammatory markers (NO, IL-1β, TNF-α, MMP-3) declined, whereas SOD and TGF-β1 levels rose, with the LESWT group exhibiting more pronounced changes (*p* < 0.01). The occurrence of adverse events showed no significant difference between the groups (*p* > 0.05). LESWT demonstrates significant efficacy in alleviating pain and reducing inflammatory markers in patients with KOA, making it a promising therapeutic option deserving of further clinical consideration.

**Trial registration**: The study protocol was registered on Chinese Clinical Trial Registry, ChiCTR2457249805. Registered 03/08/2022, https://www.chictr.org.cn/.

## Introduction

Knee osteoarthritis (KOA) is a degenerative joint condition commonly affecting middle-aged and older individuals. This disease is characterized by the breakdown of cartilage, hardening of the bone beneath the cartilage, thickening of the synovial membrane, bone spur development, and tightening of soft tissues. These changes lead to pain, limited mobility, joint deformities, and other complications, significantly impacting patients’ quality of life and contributing to high rates of disability^1,2^.

For advanced cases of KOA, surgical interventions such as knee replacement are often recommended. However, in the early and intermediate stages, non-surgical approaches like medication, physical therapy, and targeted exercises are typically employed to alleviate symptoms and slow disease progression^1,3,4^.

Low-intensity pulsed ultrasound (LIPUS) is a widely used physical treatment in physiotherapy. It involves the use of low-frequency sound waves (typically in the range of 1–3 MHz) applied to body tissues to produce primarily mechanical effects rather than thermal effects^5^. These mechanical effects are designed to promote soft tissue repair, reduce inflammation, improve blood circulation, enhance cellular metabolic activity, and alleviate pain^6^. Additionally, studies suggest that LIPUS may aid in the regeneration of joint cartilage in animal models with cartilage damage^7–9^. However, the evidence for cartilage regeneration in humans remains limited and requires further research^10^. Extracorporeal shock wave therapy (ESWT) was initially developed for treating urinary calculi, but has since been extended to address musculoskeletal conditions^11,12^. High-energy ESWT is typically employed for conditions like bone nonunion and avascular necrosis of the femoral head^13^, while low-energy ESWT is more commonly used to manage soft tissue pain disorders, such as plantar fasciitis^14^ and tennis elbow^15^. In addition to these indications, ESWT has also been applied in the management of KOA, where it has been reported to alleviate pain, improve joint function, and support cartilage protection. Clinical studies and meta-analyses suggest that ESWT provides short-term benefits in KOA patients, making it a non-invasive therapeutic option of growing clinical relevance^16,17^.

Unlike LIPUS, which primarily modulates inflammatory cytokines and promotes cartilage repair, ESWT has been shown to reduce peripheral nerve sensitivity, stimulate subchondral bone remodeling, and enhance chondrocyte activity. These complementary mechanisms provide a rationale for combining both treatments to achieve broader therapeutic effects in KOA^17–19^.

Numerous studies have demonstrated that LIPUS can effectively slow the progression of knee osteoarthritis (KOA) by suppressing the expression of inflammatory markers such as IL-1β, TNF, MMP-3, TIMP-1, and TGF-β1 in cartilage and synovial tissue, as well as regulating free radical metabolism in the bloodstream^20^. This helps reduce joint inflammation, improve blood flow, alleviate muscle spasms, enhance joint mobility, and provide pain relief^8,10^.

However, the development of KOA involves complex mechanisms, and single-treatment approaches often have limitations. In this study, we explored the use of combined LIPUS and extracorporeal shock wave therapy (LESWT) for KOA patients. We evaluated its impact on pain relief and inflammatory factor levels, while also comparing its effectiveness and safety to LIPUS combined with ESWT. This approach aims to expand the range of treatment options available for KOA.

## Materials and methods

### Subjects

**Fig. 1:**
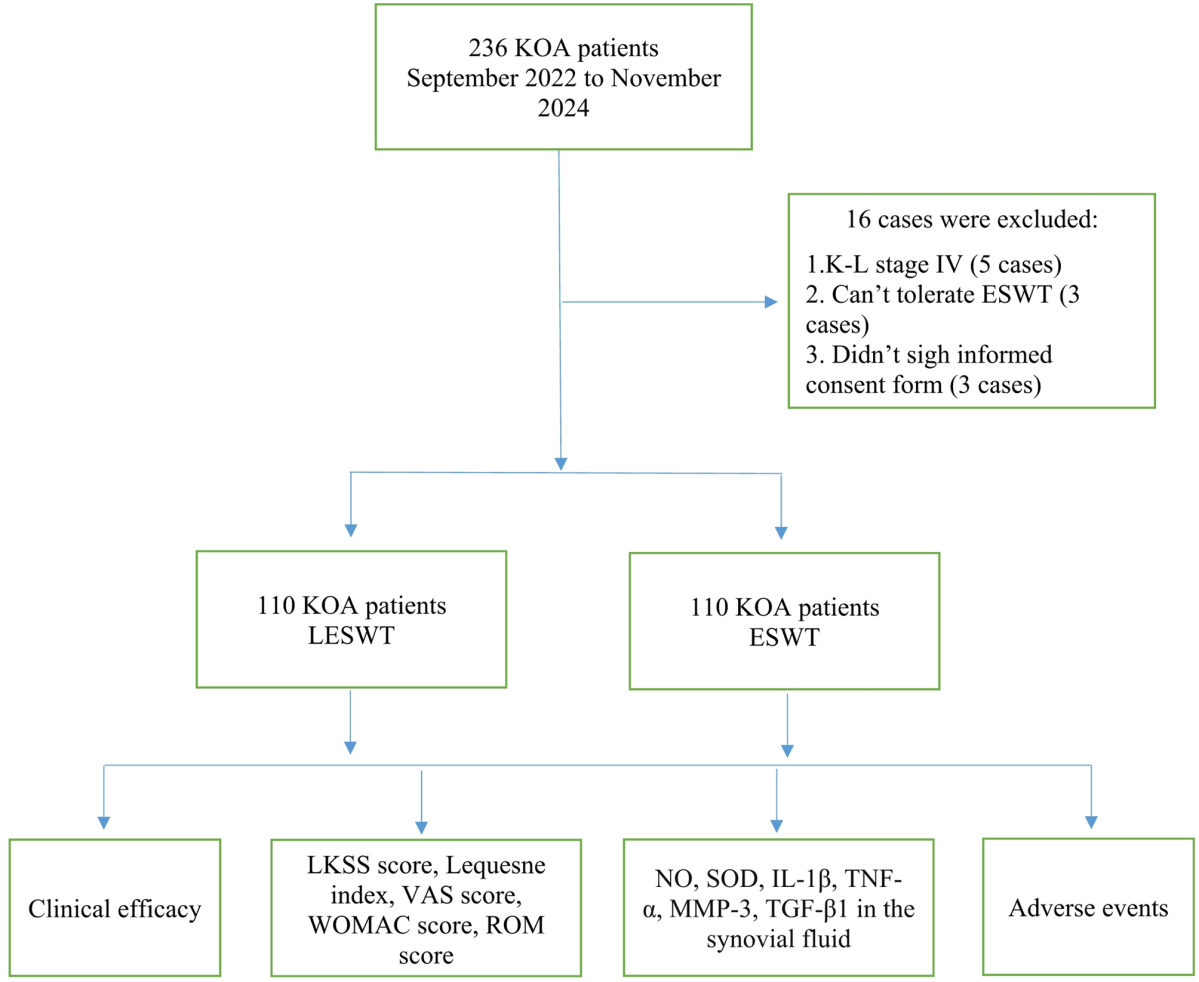
The flow of the assay. KOA: knee osteoarthritis. ESWT: extracorporeal shock wave therapy. LESWT: Low-intensity pulsed ultrasound combined with extracorporeal shock wave therapy. LKSS: lysholm kness. VAS: visual analogue scale. WOMAC: Western Ontario and McMaster Universities Arthritis Index. ROM: range of motion. NO: nitric oxide. SOD: superoxide dismutase. IL-1β: Interleukin-1β. TNF-α: tumor necrosis factor α. MMP-3: matrixmetalloproteinase-3. TGF-β1: transforming growth factor-β1.

This study is a randomized controlled trial approved by the ethics committees at Al-Baath Hospital and the Faculty of Medicine at Al-Baath University (Approval No: Al-B 77-04890). It is registered under the Clinical Trial Registry number ChiCTR2457249805 and was designed in accordance with the CONSORT 2010 statement. Between September 2022 and November 2024, 236 patients with KOA were screened from eight Rehabilitation and Orthopaedic centers across four cities in Syria: Damascus, Aleppo, Homs, and Latakia (two centers per province). Of these, 16 patients were excluded: eight participants were ineligible (did not fulfill the inclusion criteria), three refused consents, and five were lost to contact. The inclusion criteria required patients to meet the American College of Rheumatology (ACR) diagnostic guidelines^21,22^, have KOA classified as Kellgren-Lawrence (K-L) stage I–III^23^, demonstrate adequate heart, lung, liver, and kidney function to tolerate ESWT, refrain from medications or knee surgery in the past three months, and provide signed informed consent. The exclusion criteria included K-L stage 0 or IV, other types of arthritis (e.g., rheumatoid, traumatic, or gouty arthritis), knee wounds, infections, tuberculosis, osteomyelitis, vascular or neurological disorders, severe varus deformity, major systemic diseases (e.g., liver, kidney, cardiovascular, or hematopoietic issues), severe osteoporosis, pregnancy, breastfeeding, inability to tolerate ESWT, insufficient knee joint fluid for testing, or any condition that could affect inflammatory factor levels. The study design is illustrated in Fig. 1.

### Randomization procedures

Patients were randomly assigned, with equal distribution (1:1 ratio), into two groups. A person not clinically involved in the study prepared opaque, sequentially numbered randomization envelopes containing the treatment allocations. Upon providing consent, each patient selected one envelope, and the assigned therapy was administered based on the allocation inside^24^.

### Therapeutic interventions

Two physiotherapists, each with over five years of clinical experience, performed the LIPUS and ESWT interventions. Prior to the trial, both therapists underwent training on the treatment protocol and met the required standards.

*LIPUS*: A conductive aqueous gel was used as the medium. LIPUS was applied in circular motions along the tibiofemoral and patellofemoral borders, targeting the lateral and medial joint margins. The treatment settings consisted of a frequency of 1 MHz, an intensity of 0.5 W/cm², a pulsed mode (1:4). These parameters were chosen based on prior clinical and preclinical studies that have demonstrated their safety and efficacy in KOA management. A frequency of 1 MHz ensures sufficient penetration into periarticular and intra-articular tissues, while intensities between 0.3 and 0.8 W/cm² have consistently been shown to reduce inflammatory cytokines (IL-1β, TNF-α, MMP-3) and stimulate cartilage repair without causing thermal injury^5,8,10,16^. The pulsed duty cycle (1:4) was used to minimize thermal effects while maximizing mechanical stimulation, which enhances chondrocyte metabolism, extracellular matrix synthesis, and anti-inflammatory effects^7,8,16^^7,8,20^. A 5 cm diameter probe was used for 20-minute sessions (Sonopuls 590, Enraf Nonius BV, The Netherlands). Treatments were administered once daily, five days a week, for five weeks.

*ESWT*: The treatment focused on localized pain points around the affected knee joint. Using an extracorporeal shock wave therapy device (MASTERPULS MP100, STORZ, Switzerland), shock wave energy was applied to the target area at 10 Hz^25^ Each treatment site received 2000 shocks at a frequency of 10 Hz. per site. The number of shocks, frequency, treatment sites, and total sessions were fixed for all patients, ensuring consistency of the intervention. The pressure level (bar) was initially set at 3 bars and was adjusted, when necessary, within a narrow range of 1.5–3 bars according to each participant’s pain tolerance, while the number of shocks per site remained constant^21^. During treatment, patients were positioned in a supine position with the knee slightly flexed (approximately 30°) and supported by a cushion to allow relaxation of periarticular tissues. The ESWT transducer was placed perpendicularly to the skin surface at the medial and lateral joint lines of the affected knee, corresponding to the primary sites of pain and tenderness identified by palpation. The path of shock wave delivery was directed toward the joint space along these lines. Targeting was based on clinical examination and palpation of tender points; no imaging guidance was used.

In the combined treatment group (LESWT), LIPUS was administered daily, five days per week, and ESWT was performed once weekly. On the days when both treatments were delivered, LIPUS was applied first, immediately followed by ESWT in the same treatment session.

### Laboratory test indicators

Before and after treatment, joint fluid was collected from patients. The nitrate reductase method was applied to assess the expression levels of nitric oxide (NO)^26^. To quantify the levels of IL-1β (#ab214025, Abcam, Cambridge, UK), TNF (#ab181421, Abcam, Cambridge, UK), TGF-β1 (#ab100647), and MMP-3 (#ab269371, Abcam, Cambridge, UK), enzyme-linked immunosorbent assay (ELISA) was used. In addition, superoxide dismutase (SOD) levels were measured using the xanthine oxidase method^27^.

### Evaluation indicators

Clinical efficacy was evaluated based on four criteria: joint pain, joint swelling, joint mobility, and walking function. For joint pain, scores were: 0 (no change), 1 (relief), 2 (significant relief), and 3 (pain disappearance). For joint swelling, scores were: 0 (no change), 1 (relief), and 2 (swelling resolved). For joint mobility, scores were: 0 (no change), 1 (improvement), and 2 (return to normal). For walking function, scores were: 0 (no change), 1 (improvement), 2 (significant improvement), and 3 (return to normal). The total score (0–10) determined efficacy: 0–1 (ineffective), 2–5 (effective), 6–8 (significant effect), and 9–10 (clinical recovery). The clinical effectiveness rate was calculated as (clinical recovery + significant effect + effective) ÷ total number of patients in each group × 100%. Between-group differences in effectiveness rate were analyzed using the chi-square test and between-group differences were analyzed using the chi-square test^28^.

Additional assessments included the LKSS score, which evaluated knee symptoms and daily function (0–100 points; higher scores indicate better function)^28^. The Lequesne Index assessed pain/discomfort, walking distance, and daily activity (0–24 points; higher scores indicate worse symptoms)^29^. The VAS score allowed patients to mark their pain level on a 100 mm scale (0 = no pain, 100 = worst pain)^30^. The WOMAC scale measured pain, stiffness, and joint function (0–96 points; higher scores indicate worse function)^31^. Range of motion (ROM) was measured using a MicroFET3 device, with patients lying prone and ROM recorded during knee flexion^32^.

### Sample size calculation

The sample size was calculated using the comparison method for two independent sample rates^28^. aiming to ensure sufficient power for detecting significant differences between groups and minimizing type II errors. The calculation considered effect size, significance level (α = 0.05), and statistical power (at least 80%).

### Statistical analysis

Continuous variables (age, BMI, LKSS, VAS, Lequesne index, WOMAC, ROM) were expressed as mean ± standard deviation (SD). Between-group comparisons of continuous variables (LESWT vs. ESWT) were performed using the independent-samples t-test. Categorical variables (e.g., sex distribution, Kellgren–Lawrence classification categories, clinical effectiveness rates) were expressed as frequencies [n (%)] and compared using the chi-square test. Since the study included only two groups, ANOVA was not required. Statistical analysis was carried out using SPSS version 26.0 (IBM Corp., Armonk, NY, USA). Statistical significance was set at *p* < 0.05.

## Results

### Clinical data

Table 1 outlines the clinical characteristics of KOA patients assigned to the LESWT and ESWT groups. In the LESWT group, 59.09% of the participants were male, with their ages spanning from 35 to 61 years, resulting in an average age of 53.68 ± 14.08 years. Similarly, in the ESWT group, males accounted for 52.73% of the participants, with their ages ranging from 34 to 65 years, leading to an average age of 56.06 ± 10.35 years. A statistical comparison between the two groups revealed no significant differences in various demographic and clinical parameters before treatment. These parameters included age distribution, gender ratio, body mass index (BMI), Lysholm knee score, Lequesne index, visual analogue scale (VAS) score, Western Ontario and McMaster Universities Osteoarthritis Index (WOMAC), range of motion (ROM), and Kellgren-Lawrence (KL) classification (*p* > 0.05). This indicates that both groups were comparable at baseline, ensuring a balanced comparison in evaluating the effects of treatment.

**Table 1:** General information of the LESWT group and the ESWT group.

	LESWT(*n* = 110)	ESWT(*n* = 110)	*p*-value
Age(years, mean ± SD)	53.68 ± 14.08	56.06 ± 10.35	0.61
Gender [n(%)]			0.63
Male	65 (59.09%)	58 (52.73%)	
Female	47 (42.71%)	52 (47.27%)	
Body mass index (kg/m^2^, mean ± SD)	23.08 ± 2.53	24.14 ± 3.63	0.73
LKSS (score, mean ± SD)	47.21 ± 8.53	47.31 ± 8.01	0.83
Lequesne index (score, mean ± SD)	15.80 ± 2.05	15.73 ± 2.42	0.73
VAS (score, mean ± SD)	7.27 ± 1.85	7.37 ± 1.53	0.83
WOMAC (score, mean ± SD)	45.35 ± 5.14	45.97 ± 6.44	0.49
ROM (°, mean ± SD)	88.45 ± 4.66	90.56 ± 6.06	0.51
K-L grade [n(%)]			0.61
I	27 (24.54%)	24 (21.72%)	
II	63 (57.26%)	57 (51.83%)	
III	20 (18.12%)	29 (26.45%)	

SD, standard deviation. LKSS, Lysholm Kness. ROM, range of motion. K-L, Kellgren-Lawrence. VAS, visual analogue scale. WOMAC, Western Ontario and McMaster Universities Arthritis Index. LESWT, LIPUS combined with extracorporeal shock wave therapy. ESWT, extracorporeal shock wave therapy.

### Clinical efficacy

Following treatment, a total of 18 individuals in the LESWT group achieved complete clinical recovery, indicating full resolution of their symptoms. Additionally, 52 participants exhibited a marked improvement in their condition, while 26 experienced moderate clinical benefits. However, 14 individuals in this group showed no noticeable changes in their symptoms. On the other hand, in the ESWT group, only 9 participants reached full clinical recovery, while 25 demonstrated significant progress in their condition. Furthermore, 47 individuals displayed moderate clinical effects, whereas 29 did not exhibit any meaningful improvement. A statistical comparison between the two groups revealed that the overall clinical effectiveness rate in the LESWT group was 87.3%, which was significantly higher than the 73.6% observed in the ESWT group. This difference was statistically significant, with a p-value of less than 0.01, indicating a clear advantage of LESWT over ESWT in terms of treatment efficacy (Table 2).

**Table 2:** Comparison of clinical efficacy between the LESWT group and the ESWT group.

	Clinical recovery	Significant effect	Effective	Ineffective	Effectiveness
LESWT (*n* = 110)	18 (16.61%)	52 (47.24%)	26 (23.53%)	14 (12.62%)	87.3%
ESWT (*n* = 110)	9 (8.18%)	25 (22.75%)	47 (42.71%)	29 (26.36%)	73.6%
*p*-value					< 0.01

LESWT, LIPUS combined with. ESWT, extracorporeal shock wave therapy. LIPUS, Low-intensity pulsed ultrasound.

### LKSS score

Table 3 provides a comprehensive overview of the overall LKSS rating along with the evaluations of specific contributing factors both before and after treatment. Following the intervention, both the ESWT and LESWT groups demonstrated statistically significant enhancements in their total LKSS scores, indicating overall functional improvement. Additionally, notable progress was observed in various key aspects, including the severity of limping, reliance on assistive devices such as canes or crutches, occurrences of joint locking, sensations of instability or giving way, intensity of pain, degree of swelling, ability to climb stairs, and capacity to perform squatting movements (*p* < 0.05). However, a comparative analysis between the two treatment groups revealed that participants in the LESWT group experienced significantly greater advancements across all assessed factors, including the total LKSS score, when compared to those in the ESWT group. These differences were statistically significant (*p* < 0.05), further reinforcing the superior efficacy of LESWT in enhancing knee function and reducing symptoms associated with KOA.

**Table 3:** Comparison of LKSS scores between ESWT group and LESWT group before and after treatment.

	ESWT (*n* = 110)		UESWT (*n* = 110)	
	Before treatment	After treatment	Before treatment	After treatment
Total score	47.56 ± 8.79	57.89 ± 7.36^a^	46.89 ± 9.57	68.35 ± 8.05^ab^
Limp	3.34 ± 1.84	4.15 ± 1.47^a^	4.23 ± 1.56	4.85 ± 0.54^ab^
Using cane or crutches	4.03 ± 1.45	4.23 ± 1.73^a^	4.15 ± 1.47	4.37 ± 1.42^ab^
Locking sensation in the knee	9.64 ± 3.12	10.84 ± 4.56^a^	9.63 ± 3.57	14.09 ± 2.93^ab^
Giving way sensation from the knee	10.72 ± 4.09	11.64 ± 4.77^a^	10.85 ± 3.95	14.83 ± 5.21^ab^
Pain	4.65 ± 1.94	8.45 ± 4.12^a^	4.82 ± 2.05	13.42 ± 4.57^ab^
Swelling	4.51 ± 1.87	6.72 ± 2.56^a^	5.27 ± 1.72	8.94 ± 2.41^ab^
Climbing stairs	5.67 ± 2.92	6.56 ± 3.43^a^	5.42 ± 3.71	8.38 ± 2.94^ab^
Squatting	2.56 ± 1.62	3.75 ± 1.72^a^	2.49 ± 1.47	3.98 ± 0.69^ab^

LESWT, LIPUS combined with extracorporeal shock wave therapy. ESWT, extracorporeal shock wave therapy. LIPUS, Low-intensity pulsed ultrasound. ^a^*p <* 0.05 before treatment. ^b^*p <* 0.05, compared with ESWT group.

### Lequesne index score

Table 4 presents a detailed comparison of the Lequesne index scores for both the ESWT and LESWT groups before and after undergoing treatment. Prior to the intervention, statistical analysis revealed no significant differences between the two groups in terms of their overall Lequesne index scores, pain and discomfort levels, maximum walking distance, or daily activity performance (*p* > 0.05), indicating comparable baseline characteristics. However, following treatment, both groups exhibited marked improvements, as reflected in significant reductions in their total Lequesne index scores. Additionally, noticeable declines were observed in the severity of pain and discomfort, limitations in walking distance, and restrictions in daily activities, with all improvements reaching statistical significance (*p* < 0.05). Despite these positive changes in both groups, further analysis demonstrated that the LESWT group achieved significantly greater reductions in all measured parameters compared to the ESWT group, highlighting its superior effectiveness in alleviating KOA symptoms (*p* < 0.05). These findings suggest that LESWT provides more pronounced benefits in reducing disease severity and enhancing functional capabilities in individuals with KOA.

**Table 4:** Comparison of Lequesne index between ESWT group and LESWT group before and after treatment.

	ESWT (*n* = 110)		LESWT (*n* = 110)	
	Before treatment	After treatment	Before treatment	After treatment
Total score	15.76 ± 1.38	12.24 ± 2.54^a^	15.72 ± 1.78	8.90 ± 2.36^ab^
Pain or discomfort	6.02 ± 1.41	4.95 ± 1.47^a^	6.28 ± 1.47	3.16 ± 0.92^ab^
Maximum distance walked	6.12 ± 1.03	4.83 ± 1.93^a^	6.15 ± 1.35	3.65 ± 1.57^ab^
Daily activity	5.73 ± 0.94	3.68 ± 1.68^a^	5.69 ± 0.93	2.69 ± 1.34^ab^

LESWT, LIPUS combined with ESWT. ESWT, extracorporeal shock before treatment. LIPUS, Low-intensity pulsed ultrasound. ^a^*p <* 0.05 before treatment. ^b^*p <* 0.05, compared with ESWT group.

### VAS, WOMAC, and ROM scores

Table 5 provides a comprehensive comparison of the VAS, WOMAC, and ROM scores for KOA patients before and after undergoing treatment. The post-treatment evaluation demonstrated a significant improvement in ROM (*p* < 0.01), alongside a marked reduction in both VAS and WOMAC scores (*p* < 0.01), highlighting a decrease in pain levels and an enhancement in joint function for both groups. However, when assessing the magnitude of these improvements, patients in the LESWT group experienced a more substantial increase in ROM and a more pronounced decline in VAS and WOMAC scores compared to those in the ESWT group. These differences were statistically significant (*p* < 0.01), indicating that LESWT yields superior benefits in alleviating pain and improving functional mobility in KOA patients compared to ESWT alone.

**Table 5:** Comparison of VAS, WOMAC and ROM scores between the UESWT group and the ESWT group.

	VAS		WOMAC		ROM	
	Before treatment	After treatment	Before treatment	After treatment	Before treatment	After treatment
LESWT(*n* = 110)	5.85 ± 1.04	2.24 ± 0.87	43.85 ± 6.86	28.27 ± 3.41^ab^	88.47 ± 4.85	124.27 ± 14.48^ab^
ESWT(*n* = 110)	6.05 ± 1.21	4.21 ± 1.41^a^	44.64 ± 7.03	36.27 ± 4.31^a^	90.53 ± 5.85	107.53 ± 15.05^a^

VAS, visual analogue scale. WOMAC, Western Ontario and McMaster Universities Arthritis Index. ROM, range of motion. LESWT, LIPUS combined with ESWT. ESWT, extracorporeal shock wave therapy. LIPUS, Low-intensity pulsed ultrasound. ^a^*p <* 0.05, compared with before treatment. ^b^*p <* 0.05, compared with ESWT group.

### Inflammatory markers in synovial fluid

**Fig. 2:**
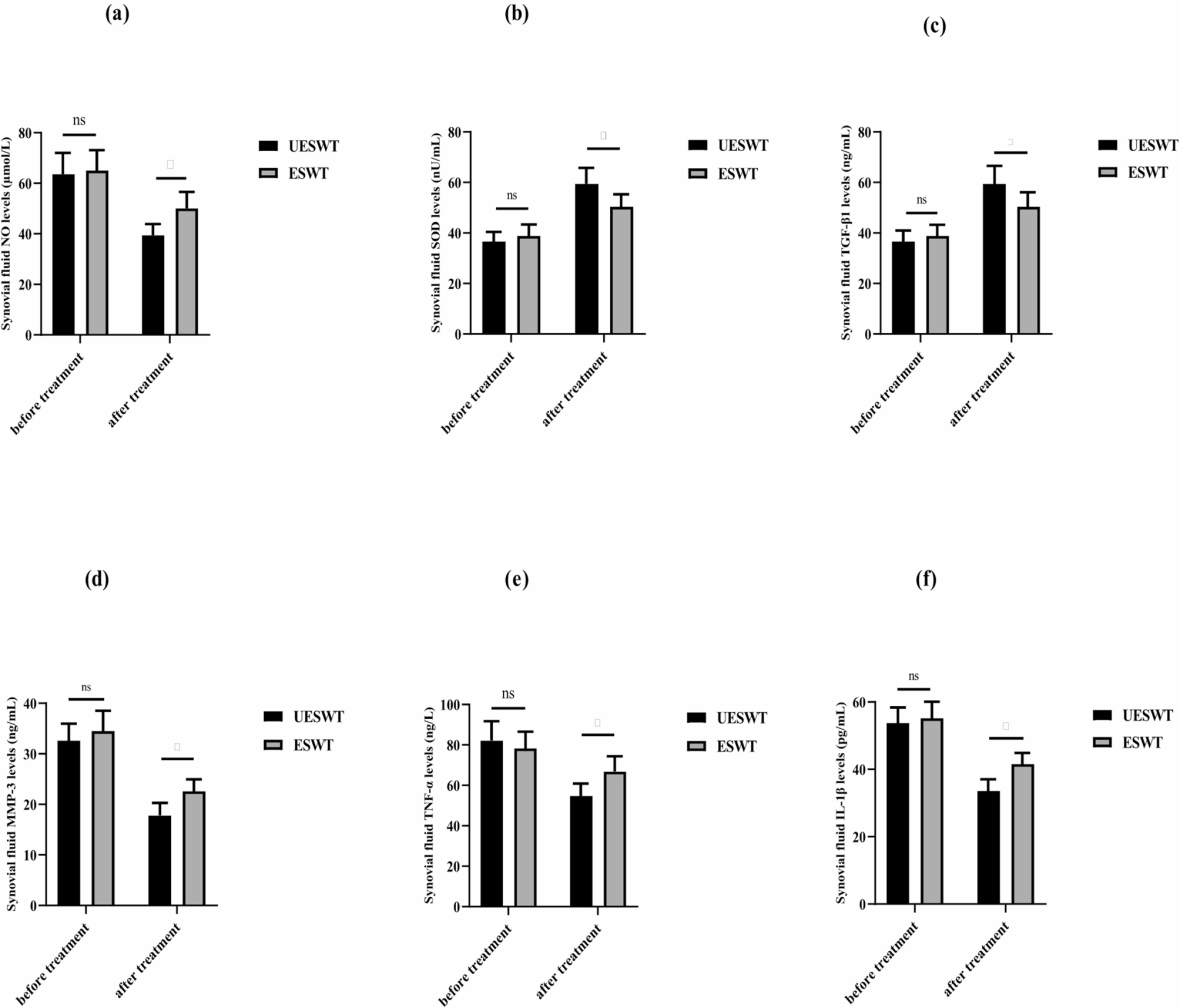
Changes in the expression levels of NO, SOD, IL-1β, TNF-α, MMP-3 and TGF-β1 in synovial fluid before and after treatment. a–f: NO, SOD, TGF-β1, MMP-3, TNF-α, and IL- 1β, respectively. *p < 0.05, compared with ESWT group. UESWT, ultrasound combined with extracorporeal shock wave therapy. ESWT, extracorporeal shock wave therapy. NO: nitric oxide. SOD: superoxide dismutase. IL-1β: Interleukin-1β. TNF-α: tumor necrosis factor α. MMP-3: matrix metalloproteinase-3. TGF-β1: transforming growth factor-β1.

The concentrations of nitric oxide (NO), superoxide dismutase (SOD), interleukin-1β (IL-1β), tumor necrosis factor-α (TNF-α), matrix metalloproteinase-3 (MMP-3), and transforming growth factor-β1 (TGF-β1) in synovial fluid were assessed before and after treatment, as depicted in Fig. 2. Post-treatment analysis revealed a notable decline in the levels of NO (Fig. 2a), MMP-3 (Fig. 2d), TNF-α (Fig. 2e), and IL-1β (Fig. 2f) in both groups, while SOD (Fig. 2b) and TGF-β1 (Fig. 2c) levels showed a significant increase (*p* < 0.01). When comparing changes relative to baseline values, patients in the LESWT group demonstrated more substantial shifts in NO, SOD, IL-1β, TNF-α, MMP-3, and TGF-β1 levels than those in the ESWT group. These variations between the two treatment groups were statistically significant (*p* < 0.01), suggesting that LESWT had a greater regulatory effect on these biochemical markers associated with inflammation and tissue remodeling in KOA patients.

### Adverse events

The most commonly reported adverse events during the treatment period included localized swelling, puffiness, pain, and joint stiffness, as detailed in Table 6. A statistical assessment comparing the incidence of these side effects between the LESWT and ESWT groups demonstrated no significant differences (*p* > 0.05). This finding indicates that both treatment approaches were generally well tolerated, with a similar safety profile and no notable discrepancies in the frequency or severity of adverse reactions.

**Table 6:** Comparison of adverse events between the study group and the control group.

	LESWT (*n* = 110)	ESWT (*n* = 110)	*p* value
Joint swelling	3 (2.72%)	4 (3.63%)	0.73
Joint puffiness	3 (2.72%)	5 (4.54%)	0.62
Joint pain	4 (3.63%)	7 (6.36%)	0.51
Joint stiffness	2 (1.81%)	4 (3.63%)	0.56

LESWT, LIPUS combined ESWT. ESWT, extracorporeal shock wave therapy. LIPUS, Low-intensity pulsed ultrasound.

## Discussion

Knee osteoarthritis is closely associated with various cytokines, which play a significant role in disease severity and progression^33^. The levels of these cytokines in the synovial fluid of the knee joint are directly linked to the progression of KOA^33^. These cytokines can be categorized into two groups: decomposing factors and synthetic factors. Decomposing factors include NO, IL-1β, TNF-α, and MMP-3, among others^34–37^. IL-1β, a potent inflammatory mediator found in various tissue cells, plays a critical role in KOA pathogenesis. It upregulates Wnt-5 A protein through the JNK pathway, enhances MMP-3 gene expression, and accelerates the degradation of the articular cartilage matrix^38^. Additionally, IL-1β suppresses the synthesis of type II collagen and aggrecan, key components of the extracellular cartilage matrix. This makes IL-1β a central factor in cartilage destruction and matrix degradation in KOA, as well as a key driver of inflammation-mediated processes in the disease^39^. Furthermore, IL-1β stimulates the production of nitric oxide synthase in cartilage and synovium, leading to elevated NO levels, which are closely associated with inflammation progression and outcomes. High NO concentrations in synovial fluid trigger oxidative stress, inhibit chondrocyte proliferation, and promote chondrocyte apoptosis, accelerating osteoarthritis progression^39^. Similarly, TNF-α plays a significant role in cartilage remodeling in KOA, with its levels correlating with disease severity and serving as a diagnostic marker^40^. Studies, such as those by Yue et al.^41^, have demonstrated that reducing TNF-α and MMP-3 levels in articular cartilage improves motor function in KOA patients. On the other hand, SOD, a cytokine that scavenges oxygen free radicals, protects cartilage cells and the extracellular matrix from oxidative damage, thereby slowing KOA progression^42,43^. The balance between these factors is crucial for maintaining knee joint cartilage stability. Disruption of this balance leads to cartilage degradation and destruction, ultimately resulting in KOA.

In recent years, ESWT has emerged as a promising treatment for KOA. Studies in both cellular and animal models have shown that ESWT stimulates cartilage repair by promoting chondrocyte proliferation, inhibiting apoptosis, and delaying cartilage degeneration^16,44^. ESWT has also been found to downregulate inflammatory factors in chondrocytes^18,19,45^, reduce NO levels in synovial fluid, and inhibit caspase-3 expression, thereby slowing osteoarthritis progression^46^. In this study, combined LIPUS and ESWT (LESWT) demonstrated superior clinical efficacy compared to (ESWT) alone. LESWT significantly reduced levels of NO, IL-1β, TNF-α, and MMP-3 in synovial fluid, delayed OA progression, and promoted cartilage cell proliferation and repair. Additionally, LESWT increased SOD and TGF-β1 expression, mitigating inflammatory damage to cartilage and supporting cartilage regeneration. Patients treated with LESWT showed significant improvements in KLSS scores, range of motion (ROM), and reductions in Lequesne index, VAS, and WOMAC scores, indicating enhanced pain relief and joint function. Researchers have suggested that ESWT reduces peripheral nerve sensitivity, inhibits pain-related factors like calcitonin gene-related peptide, and decreases substance P release, thereby increasing pain thresholds and providing effective analgesia^47,48^. These findings align with previous studies demonstrating ESWT’s short-term benefits in pain relief and functional improvement for KOA patients^17^. Furthermore, LIPUS has been shown to activate the nervous system, desensitize peripheral nociceptors^49^, and reduce pro-inflammatory cytokines, contributing to its analgesic and anti-inflammatory effects^50^. The combination of ultrasound with ESWT enhances these benefits, making LESWT a more effective treatment for KOA.

LIPUS, in particular, has shown promise in KOA management. It not only alleviates pain but also improves muscle spasms, reduces local edema, and enhances tissue excitability^51^. The mechanical and thermal effects of ultrasound can penetrate deep into tissues, promoting blood flow and cellular repair^52^. In this study, continuous wave ultrasound was used, but further research is needed to explore the effects of density and discontinuous waves. The safety profile of ultrasound therapy is excellent, with minimal adverse effects reported, making it a viable option for long-term KOA management. However, the first limitation of our study is that optimizing LESWT for KOA treatment remains challenging. Parameter settings, such as penetration depth, waveform, energy flow density, and treatment frequency, are critical for achieving optimal outcomes. High-energy ESWT, while effective for conditions like nonunion fractures and urinary calculi, can cause tissue damage and is less suitable for KOA. In contrast, low- to medium-energy ESWT is safer and more commonly used for KOA, as it minimizes tissue damage and requires shorter recovery times. Further research is needed to determine the ideal energy flux density for different patient populations.

The second limitation of this study is the absence of a LIPUS-only treatment group. Thus, while our findings confirm that combining ESWT with LIPUS improves outcomes compared to ESWT alone, they do not establish whether ESWT adds benefit beyond LIPUS monotherapy. Previous studies have shown that LIPUS alone reduces pain and stiffness and enhances functional outcomes in KOA. For instance, Zhou et al.^10^ reported significant improvements in WOMAC and VAS scores in a meta-analysis of randomized trials, while Rothenberg et al.^8^ and Yang et al.^7^ demonstrated cartilage repair in preclinical models through modulation of IL-1β, TNF-α, and MMP-3. These findings align with our biochemical results, though the magnitude of improvement in our study suggests a potential additive or synergistic effect of ESWT.

Most clinical trials on LIPUS employed a frequency of 1–3 MHz and intensity of 0.3–0.8 W/cm² in pulsed mode for 15–20 min^5,10^. Our protocol (1 MHz, 0.5 W/cm², pulsed mode, 20 min) was consistent with these parameters, and patient groups were generally similar (Kellgren–Lawrence stage II–III). Direct comparisons of ESWT and LIPUS are limited, but existing evidence suggests ESWT primarily influences subchondral bone remodeling and nociceptive signaling^18,19^, while LIPUS mainly modulates inflammatory mediators and cartilage repair^8,20^. The superior improvements with LESWT in our study may therefore arise from their complementary mechanisms.

The third limitation of this trial is the lack of a sham LIPUS intervention in the ESWT group. Without a placebo control, it cannot be fully excluded that some of the improvements seen in the LESWT group may have been influenced by non-specific effects, such as patient expectations or placebo responses. Future randomized trials should incorporate sham LIPUS conditions alongside active ESWT and combined therapy to more accurately distinguish the specific therapeutic contributions of each modality.

Moreover, the fourth limitation of our study is the absence of imaging techniques (such as radiography, MRI, or ultrasonography) to assess structural changes in the knee joint. It relied on validated clinical outcome measures and biochemical markers, which provide solid evidence of symptomatic relief and inflammatory improvements but do not directly capture morphological alterations. Future research should integrate advanced imaging methods to complement clinical outcomes and offer a more comprehensive evaluation of cartilage integrity and disease progression.

Additionally, this study had a small sample size, limiting the generalizability of the findings. Future studies should include larger cohorts and investigate long-term outcomes, including changes in articular cartilage structure and function. The combination of ultrasound and ESWT holds great potential for KOA treatment, but more clinical data is needed to refine treatment protocols and maximize therapeutic benefits.

## Conclusion

Through this study, it is evident that combined LESWT addresses the limitations of single-treatment approaches for KOA patients. It significantly alleviates pain, reduces inflammatory factor levels, and improves clinical outcomes. As a result, LESWT emerges as a promising and clinically viable treatment option for KOA, deserving greater attention in therapeutic practice. Further trials with inclusion of a LIPUS-only group are needed to determine whether the observed benefits of LESWT derive from true synergy or simply reflect the established effects of LIPUS monotherapy.

## Data Availability

The datasets used and/or analysed during the current study available from the corresponding author on reasonable request.
